# *Magnaporthe*-Unique Gene *MUG1* Is Important for Fungal Appressorial Penetration, Invasive Hyphal Extension, and Virulence in Rice Blast Fungi

**DOI:** 10.3390/jof10080511

**Published:** 2024-07-23

**Authors:** Huixia Zhang, Zhiyi Chen, Zechen Yu, Liu Tang, Wenqiang Gao, Xunli Lu, Jun Yang

**Affiliations:** 1 MARA Key Laboratory of Pest Monitoring and Green Management, Department of Plant Pathology, College of Plant Protection, China Agricultural University, Beijing 100193, China; zhanghuixia@cau.edu.cn (H.Z.); zhiyichen@cau.edu.cn (Z.C.); yuzechen2003@163.com (Z.Y.); tangliu0802@162.com (L.T.); gwenqiang@outlook.com (W.G.); luxunli@cau.edu.cn (X.L.); 2 MARA Key Laboratory of Surveillance and Management for Plant Quarantine Pests, Department of Plant Biosecurity, College of Plant Protection, China Agricultural University, Beijing 100193, China

**Keywords:** rice blast fungi, *Pyricularia*, orphan gene, conidiation, pathogenesis

## Abstract

Species-unique genes that encode specific proteins and have no homologs in other species play certain roles in the evolution of species and adaptations to external environments. Nevertheless, the biological roles of unique genes in plant pathogenic fungi remain largely unknown. Here, four *Magnaporthe*-unique genes (*MUG1*–*MUG4*), which were highly expressed during the early infection stages, were functionally characterized in the rice blast fungus *Magnaporthe oryzae.* Subcellular localization assays revealed that Mug1, Mug2, and Mug4 were localized to the cytoplasm and that Mug3 was localized into the nuclei. Furthermore, through gene knockout and phenotypic analysis, only *MUG1* was found to be indispensable for fungal virulence and conidiation. Detailed microscopic analysis revealed that the deletion mutants of *MUG1* clearly exhibited reduced appressorial turgor pressure and invasive hyphal development. Taken together, our findings indicate that the *Magnaporthe*-unique gene *MUG1* plays a vital role in infection-related morphogenesis and virulence in rice blast fungi and suggest the specific and important roles of species-unique genes.

## 1. Introduction

Species-unique genes refer to proteins encoded in specific taxa that are endemic and have no homologous sequences in other species [1,2]. Compared with non-unique genes, species-unique genes are short, contain few introns and detectable domains, are moderately expressed, evolve fast, and participate in species-specific adaptation processes [3,4]. Species-unique genes are supposed to originate from two mechanisms, the duplication divergence model and the *de novo* model, and play important roles in the evolution and adaptation of organisms to external environments [5,6]. Species-unique genes might have important roles in the development of novel traits, the adaptation to new ecological niches, and the emergence of lineage-specific phenotypes [7]. Species-unique genes are also involved in species-specific biological processes and pathways and might contribute to the unique characteristics of different lineages [8,9]. Understanding their origin and function helps in unraveling the mechanisms underlying the emergence of biological diversity and the evolution of complex traits [7].

With the continuous development of sequencing technology, an increasing number of species-unique genes have been identified, which comprise 10–20% of all annotated genes in a species [1,10]. They are supposed to be responsible for the development, reproduction, and survival and to contribute to the genetic diversity and evolutionary success of a species by adapting to its external environment and ecological niche [3,11,12,13]. In pathogenic fungi, most species-unique genes have not been functionally characterized. To date, only a few have been reported. For example, *EED1*, a gene unique to *Candida albicans*, plays an important role in maintaining filamentous growth and mycelium expansion on the host surface [14]. *OSP24*, the gene unique to the wheat head scab pathogen *Fusarium graminearum*, is required for the growth of infection hyphae in wheat germ tissue, although it has no effects on mycelial growth and primary infection [2].

Presently, *Magnaporthe oryzae*, which causes devastating rice blast disease across the world, is used as a model for functional genomics among plant pathogenic fungi [15]. This fungus employs a unique structure called the appressorium to penetrate the rice plant’s leaf surface and initiate successful infection with bulbous invasive hyphae [16]. In the past two decades, hundreds of genes have been identified and functionally characterized by forward and reverse genetics approaches to this pathogen [17,18,19,20]. Nevertheless, only a few *Magnaporthe*-unique genes have been reported to play certain roles in fungal pathogenicity. In *M. oryzae*, more than a dozen of species-unique genes, including *MoSPC1-MoSPC7*, *MIR1*, and *MoHKR1*-*MoHKR7*, which are highly expressed during the infection stage, have been identified but only *MoHKR1* has been found to play crucial roles in plant infection [1,4,21]. Based on the data of the expression profile at different developmental and infection stages of *M. oryzae*, genes highly expressed at the early infection stage and playing roles during plant infection were identified [22], among which four genes unique to *Magnaporthe*, named as *MUG1-MUG4* (*Magnaporthe oryzae* unique genes) in this study, were selected for analysis. Of these four genes, *MUG1* was indispensable for fungal infection-related morphogenesis and plant infection. We also investigated the subcellular localization of these four *Magnaporthe*-unique proteins.

## 2. Materials and Methods

### 2.1. Fungal Strains and Growth Conditions

The wild-type strain P131 [23], the deletion mutants of *MUG1*, *MUG2*, *MUG3*, and *MUG4*, and their various transformants generated in this study were cultured at 28 °C on oatmeal tomato agar (OTA) plates [24]. Two-day-old mycelia shaken in a liquid complete medium (CM) at 160 rpm were collected and then used to isolate nucleic acids, proteins, and protoplasts. The isolated protoplasts were transformed using the PEG/CaCl_2_ method [25]. Media supplemented with 250 µg/mL hygromycin B (Roche, Basel, Switzerland) or 400 µg/mL neomycin (Ameresco, Framingham, MA, USA) were used to select hygromycin-resistant or neomycin-resistant transformants, respectively [26]. To assess mycelial growth and colony characteristics, fungi were cultivated on OTA plates at 28 °C for 120 h, as previously described [16]. Conidia harvested from conidiation plates were used for the virulence test and infection process observation [27].

### 2.2. Nucleic Acid Manipulation

Genomic DNAs were extracted from vegetative hyphae with the CTAB (cetyltrimethylammonium bromide) protocol [28]. Standard protocols of molecular biology were followed for plasmid isolation, Southern blotting analyses, and DNA enzymatic digestion [15]. Probes were labeled with the random primer labeling kit (TaKaRa, Kusatsu, Japan). Plasmid constructs were sequenced by the TSINGKE company (TSINGKE, Beijing, China). All primers (Supplementary Table S1) were synthesized by the TSINGKE company.

### 2.3. Generation of the MUG Gene Deletion Mutant

To generate the *MUG* gene deletion vector pkov21:MUG1, pkov21:MUG2, pkov21:MUG3, and pkov21:MUG4, the 1.2–1.5 kb upstream and downstream sequences of *MUG* genes were amplified with the paired primers listed in Supplementary Table S1 [27]. The resulting PCR products were cloned into the restriction enzyme restriction sites of pkov21 [29,30]. The *MUG1*, *MUG2*, *MUG3*, and *MUG4* gene deletion vectors pkov21:MUG1, pkov21:MUG2, pkov21:MUG3, and pkov21:MUG4 were transformed into protoplasts of P131. Hygromycin-resistant transformants were isolated and assayed for resistance to neomycin. The transformants were screened by PCR with primer pairs MUG1, MUG2, MUG3, and MUG4 Check-F/Hpt-Up and Check-R/Hpt-down (Supplementary Table S1). The putative *MUG* genes deletion mutants were further confirmed by Southern blotting.

### 2.4. Complementation Strains

For complementation strains, a fragment containing the 1.5-kb promoter region and the *MUG1* gene coding region was amplified and cloned into the plasmid pKN (Supplementary Table S1) [31]. The complementation vector was transformed into the MUG1ko1 mutant protoplast. The CM plates were supplemented with 400 µg/mL neomycin (Amresco, Famingham, MA, USA) to select the complementation transformants.

### 2.5. Construction of the Mug-eGFP Fusion Strains

Vectors were constructed for subcellular localization studies involving the *MUG1*, *MUG2*, *MUG3*, and *MUG4* genes. The *MUG1*, *MUG2*, *MUG3*, and *MUG4* genes were amplified and ligated into the C-terminus of the green fluorescent protein (GFP) gene to the pKNRG vector [29]. This vector contains the constitutive RP27 promoter, which drives the expression of pKNRG-Mugs-GFP fusion proteins. The vectors pKNRG-Mugs-GFP were generated by amplifying the *MUG* genes with the primer pair Mug-GFP F/R (Supplementary Table S1). Linearized pKNRG-Mugs-GFP was then transformed into protoplasts and selected on solid CM with 400 μg/mL neomycin. In the end, all of the resulting transformants were assayed by observing the green fluorescence signals under a confocal microscope (Nikon Fluorescence microscopy Ni90, Nikon, Tokyo, Japan) [32].

### 2.6. Plant Inoculation Assay

Four-week-old seedlings of rice cultivar ‘LTH’ and barley cultivar ‘E8’ were used for infection assays. For the infection assay, conidial suspensions with a concentration of 5 × 10^4^ conidia/mL were sprayed onto rice leaves and 2 × 10^4^ conidia/mL were sprayed onto barley leaves; alternatively, conidial suspensions with a concentration of 1 × 10^5^ conidia/mL were used to inoculate wound leaves and then incubated as previously described [24,33]. Disease lesions were examined and photographed 5–7 days post inoculation (dpi).

### 2.7. Microscopy

Conidial suspensions with a concentration of 2 × 10^5^ conidia/mL were used to inoculate barley epidermis cells; the infection process was observed at 24 and 36 h post inoculation (hpi) [34]. The samples were observed and photographed under a Nikon Ni90 fluorescence microscope [15,27]. A rectangular plug of the culture was cut from an OTA plate and placed on a glass slide. Conidiation was observed and photographed under a Nikon SMZ800N stereomicroscope (Nikon, Tokyo, Japan) [25]. A conidial suspension (5 × 10^4^ conidia/mL) was placed on a microscope cover glass under humid conditions at 28 °C in darkness and the samples were microscopically observed at intervals to measure appressorium formation [35]. The percentages of conidia forming appressoria was determined by microscopic examination of at least 100 conidia for more than three times. The appressorium turgor was measured using an incipient cytorrhysis (cell collapse) assay with 1–3 M glycerol solution [36]. The experiments were repeated three times and more than 100 appressoria were observed for each replicate.

### 2.8. Quantitative Reverse Transcription PCR (qRT-PCR)

Total RNA samples were extracted with an RNA extraction kit (Invitrogen, Waltham, MA, USA) from vegetative hyphae grown in liquid CM, conidia produced on OTA plates, appressoria formed on hydrophobic film surfaces, and infected-barley leaves inoculated with conidial suspensions [37]. The crude RNA was pretreated with DNase I (Takara) and was then reverse transcribed with a PrimeScript RT-PCR Kit (Takara). The qRT-PCR was performed on an ABI 7500 real-time PCR system (Applied Biosystems, Waltham, MA, USA) according to the manufacturer’s instructions. The Actin gene (MGG_03982) was used as an endogenous control. To compare the relative abundance of target transcripts, a mycelial sample was used as a calibrator and the average threshold cycle (Ct) was normalized to the Actin gene for each of the treated samples as described [38].

## 3. Results

### 3.1. Four Magnaporthe-Unique Genes Were Highly Expressed during the Fungal Early Infection Stage

**Figure 1:**
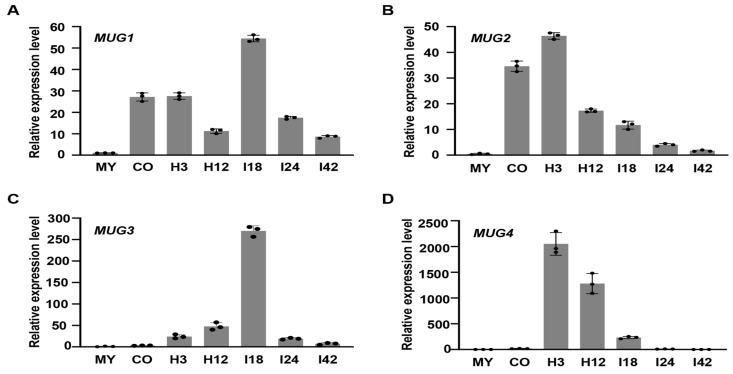
The four *MUG* genes were highly expressed at the early infection stage of *Magnaporthe oryzae.* Expression levels of the *MUG1* (**A**), *MUG2* (**B**), *MUG3* (**C**), and *MUG4* (**D**) were evaluated by qRT-PCR at different stages of fungal development, including vegetative hyphae (MY), conidia (CO), appressoria formed on hydrophobic film surfaces at 3 and 12 hpi (H3 and H12), and infected barley leaves at 18, 24, and 42 hpi (I18, I24, and I42), respectively. The expression levels of the four *MUG* genes were first normalized with the Actin gene in each tested tissue and the expression levels of *MUG* genes in MY were arbitrarily set to unity. The means and standard deviations were calculated based on two independent experiments with three replications.

The four *Magnaporthe*-unique genes *MUG1* to *MUG4* had no or only one intron, i.e., *MUG1* (MGG_12307), *MUG2* (MGG_15077), and *MUG3* (MGG_16034) were a singleton of 297, 321, and 603 bp, respectively, and *MUG4* (MGG_17927) was 319 bp with one intron. Their expression patterns were measured using qRT-PCR in samples originating from vegetative hyphae (MY), conidia (CO), appressoria formed on hydrophobic film surfaces at 3 and 12 hpi (H3 and H12), and infected barley leaves at 18, 24, and 42 hpi (I18, I24, and I42), respectively. All four genes had relatively low expression levels in MY and I42 (Figure 1A–D). Nevertheless, *MUG1* and *MUG3* had the highest expression level in I18 and *MUG2* and *MUG4* had the highest expression level in H3, all of which were over 50-fold greater than that in MY (Figure 1A–D). Moreover, *MUG1* and *MUG2* also had a higher expression level in CO, over 20-fold more than that in MY (Figure 1A,B). Overall, the four *Magnaporthe*-unique genes, *MUG1*-*MUG4*, were all highly expressed during the early infection stage of *M. oryzae*.

### 3.2. Mug1, Mug2, and Mug4 Were Localized into the Cytolasm and Mug3 Was a Nuclear Protein

To investigate the function of *MUG* genes, four gene deletion vectors were constructed by replacing the open reading frame of *MUG* genes with the hygromycin cassette and transforming into wild-type strain P131. Putative *MUG* gene deletion transformants were screened using PCR and the resulting transformants were verified by DNA gel blot. The analysis revealed that two *MUG1* gene-deletion mutants, MUG1ko1 and MUG1ko2, had longer bands (4.2 kb) than the wild-type band (1.7 kb) after *Sal*I digestion (Figure S1A). Similarly, the *MUG2* gene-deletion mutant MUG2ko1, *MUG3* gene-deletion mutant MUG3ko1, and *MUG4* gene-deletion mutant MUG4ko1 were verified by DNA gel blot analysis (Figure S1B–D).

**Figure 2:**
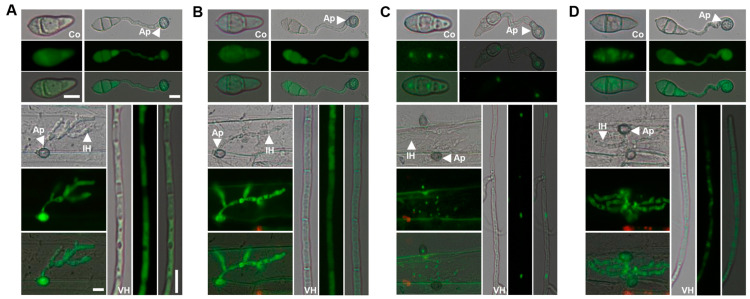
Mug1, Mug2, and Mug4, localized in the cytoplasm, and Mug3, localized in the nuclei. Subcellular localization of the Mug1–GFP (**A**), Mug2-GFP (**B**), Mug3-GFP (**C**), and Mug4-GFP (**D**) fusion proteins in conidia (Co), appressorium (Ap), invasive hyphae (IH), and vegetative hyphae (VH). Bar, 10 μm.

To determine the subcellular localization of the four Mug proteins, their N- and C-terminal fused GFP were generated and transformed into the corresponding gene knockout mutants but no GFP signals were observed at any of the developmental stages. A similar strategy was applied by constitutively expressing the GFP-fused proteins with the RP27 promoter. For the *mug1* deletion mutants, the Mug1–GFP fusion restored the defects in plant infection and conidiation. Strong GFP signals were evenly distributed in the cytoplasm of conidia (Co), appressorium (Ap), invasive hyphae (IH), and vegetative mycelia (VH) of transformants expressing Mug1–GFP (Figure 2A). Similarly, Mug2-GFP was also evenly distributed in the cytoplasm of all tissues (Figure 2B) and Mug3-GFP was concentrated in the nuclei (Figure 2C). Mug4-GFP was also a cytoplasmic protein, although it seemed unevenly distributed in conidia and vegetative mycelia (Figure 2D).

### 3.3. MUG1 Was Indispensable for Full Virulence

**Figure 3:**
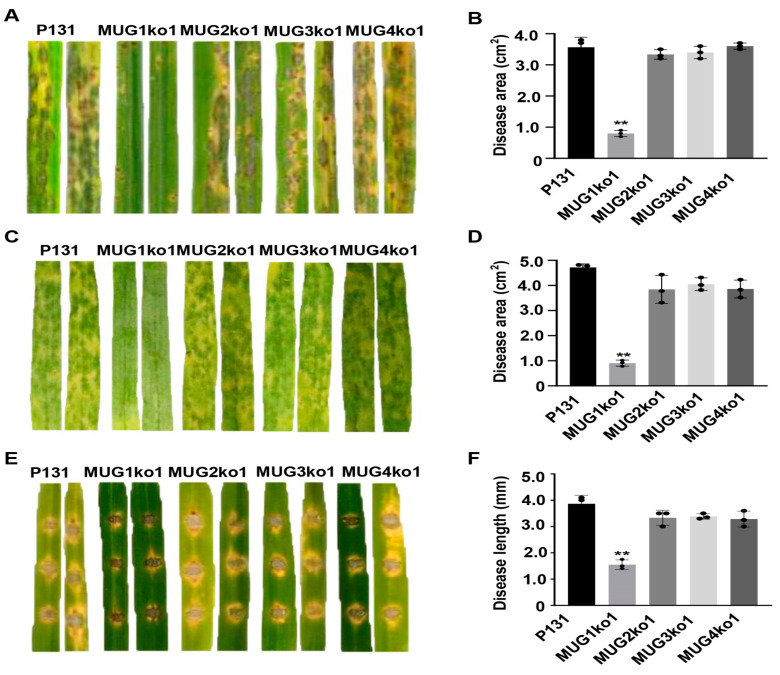
*MUG1* was indispensable for full virulence. (**A**) Typical rice leaves of the susceptible cultivar ‘LTH’ sprayed with conidia of strains P131, MUG1ko1, MUG2ko1, MUG3ko1, and MUG4ko1 photographed at 5 dpi. (**B**) Statistics on disease lesion areas formed by strains P131, MUG1ko1, MUG2ko1, MUG3ko1, and MUG4ko1 on susceptible rice cultivar ‘LTH’. Means and standard deviations were calculated from the lesions formed on the middle 5-cm of the fourth leaves. Three independent experiments were conducted with at least 10 leaves in each replicate. Asterisks indicate a significant difference at *p* < 0.01 according to a *t*-test. (**C**) The leaves of susceptible barley cultivars were sprayed with conidia of strains P131, MUG1ko1, MUG2ko1, MUG3ko1, and MUG4ko1. Photos were taken at 4 dpi. (**D**) Statistics on disease lesion areas formed by strains P131, MUG1ko1, MUG2ko1, MUG3ko1, and MUG4ko1 in (**C**). Three independent experiments were conducted with at least 10 leaves in each replicate. Asterisks indicate a significant difference at *p* < 0.01 according to a *t*-test. (**E**) The leaves of rice seedlings at the four-leaf stage were abraded and inoculated with conidia of strains P131, MUG1ko1, MUG2ko1, MUG3ko1, and MUG4ko1. Representative disease symptoms were photographed at 5 dpi. (**F**) Statistics on the length of individual disease lesions formed by strains P131, MUG1ko1, MUG2ko1, MUG3ko1, and MUG4ko1 on abraded rice leaves as described in (**E**). Means and standard deviations were calculated from three independent experiments in which at least 10 leaves were inoculated for each of the strains.

To determine whether *MUG* genes were required for plant infection, conidia of strains P131, MUG1ko1, MUG2ko1, MUG3ko1, and MUG4ko1 were sprayed onto the seedlings of susceptible rice cultivar ‘LTH’. All strains MUG2ko1, MUG3ko1, and MUG4ko1 generated comparative disease lesions with strain P131, while strain MUG1ko1 produced noticeably fewer lesions under the same condition (Figure 3A). Statistically, MUG1ko1 caused approximately 0.9 cm^2^ disease areas, on average, on 5 cm leaves, which were significantly smaller than that with 3.5 cm^2^ disease areas caused by strains P131, MUG2ko1, MUG3ko1, and MUG4ko1 (Figure 3B). Similar results were obtained by inoculating the barley seedlings (Figure 3C,D). Furthermore, when the conidia were used to inoculate abraded rice leaves, strain MUG1ko1 caused disease lesions that were 3.0 mm in length on average, compared to 7.0-mm long lesions caused by strains P131, MUG2ko1, MUG3ko1, and MUG4ko1 (Figure 3E,F). Therefore, *MUG1*, but not other *MUG* genes, were required for fungal virulence.

### 3.4. MUG1 Was Required for Appressorium Turgor Pressure and Invasive Hyphal Growth

**Figure 4:**
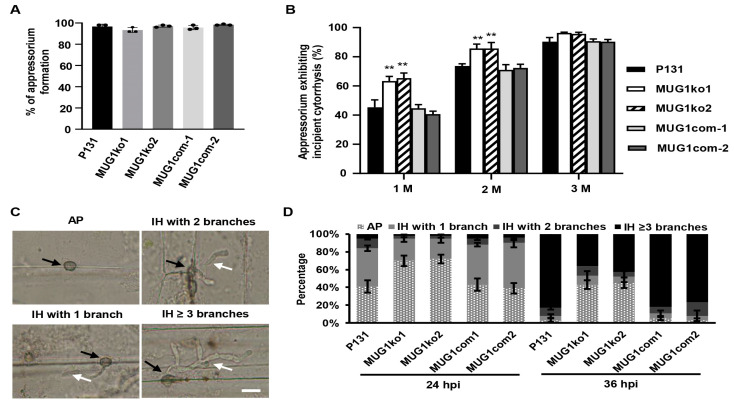
*MUG1* was required for appressorium turgor pressure and invasive hyphal growth. (**A**) Statistics on appressorium formation of wild-type strain P131, the *MUG1* gene deletion mutants MUG1ko1 and MUG1ko2, two gene complementation strains MUG1com1 and MUG1com2. The means and standard deviations were calculated based on two independent experiments with three replications. (**B**) Statistics on appressoria exhibiting incipient cytorrhysis of strains P131, MUG1ko1, MUG1ko2, MUG1com1, and MUG1com2 with 1, 2, and 3 M glycerol. The means and standard deviations were calculated based on two independent experiments with three replications. Asterisks indicate a significant difference at *p* < 0.01 according to a *t*-test. (**C**) Classification of the infection process of fungal strains in the epidermal cells of barley leaves. AP, appressoria without primary invasive hyphae (IH) or penetration pegs; IH with 1 branch and IH with primary IH or penetration pegs; IH with 2 branches and IH with 2 branches; and IH **≥** 3 branches and IH with at least 3 branches. Scale bar, 10 μm. The black arrows point to the appressoria and the white arrows point to the IH. (**D**) Statistics on the percentages of the fungal infection process of strains P131, MUG1ko1, MUG1ko2, MUG1com1, and MUG1com2 on barley leaves at 24 and 36 hpi. Means and standard deviations were calculated from three independent experiments, each with at least 100 appressoria scored.

To clarify the reason for reduced virulence by the deletion of *MUG1*, the infection processes of strains MUG1ko1 and MUG1ko2 were investigated. First, the percentages of appressorium formation by strains MUG1ko1 and MUG1ko2 were indistinguishable from those of wild-type strain P131 on hydrophobic glass cover slides (Figure 4A). Second, the internal concentration of solute and the turgor pressure within the appressoria were measured by the incipient cytorrhysis assay using glycerol [36]. At 1 M glycerol, the appressorium collapse rate was approximately around 62% in strains MUG1ko1 and MUG1ko2 compared with 40% in strain P131 (Figure 4B); similar results were obtained with 2 M glycerol (Figure 4B), suggesting that the turgor pressure in the appressoria of strains MUG1ko1 and MUG1ko2 was seriously reduced. Third, the development of invasive hyphae was investigated by using the conidia to inoculate epidermal cells of barley leaves and was divided into four stages: AP, only appressoria; invasive hyphae (IH) with 1 branch, including appressoria with penetration pegs or primary (IH); IH with two branches; and IH **≥** 3 branches, IH with at least three branches (Figure 4C). At 24 hpi, only 30% IH was observed under the MUG1ko1 and MUG1ko2 appressoria, while approximately 60% of the P131 appressoria formed branches of IH. At 36 hpi, approximately 90% of the P131 appressoria formed IH **≥** 3 branches, whereas about 40% of the MUG1ko1 and MUG1ko2 IH formed IH **≥** 3 branches (Figure 4D), signifying that the development of the IH of strains MUG1ko1 and MUG1ko2 in host plant cells was severely impaired. All above defects of strains MUG1ko1 and MUG1ko2 could be rescued in transformants MUG1com1 and MUG1com2, which expressed a wild-type *MUG1* allele in strain MUG1ko1 (Figure 4A–D). Overall, these findings suggest that *MUG1* regulates appressorial turgor pressure and invasive hyphae extension, thus contributing to the attenuated virulence of *MUG1* deletion mutants.

### 3.5. MUG1 Was Important for Conidiation

**Figure 5:**
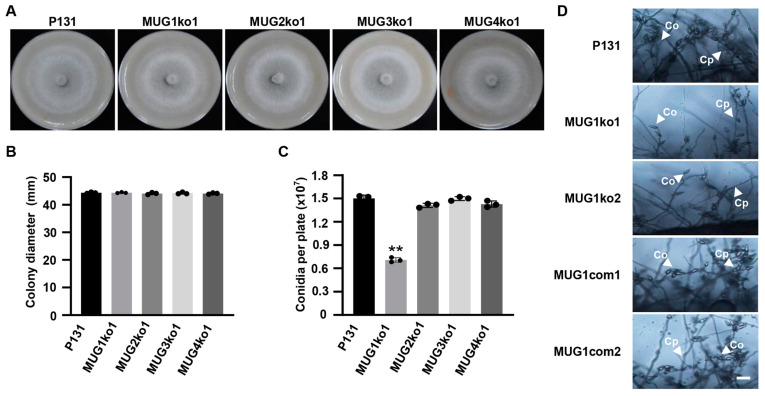
*MUG1* was required for conidiation. (A) Colony of wild-type strain P131 and gene deletion mutants MUG1ko1, MUG2ko1, MUG3ko1, and MUG4ko1 cultured on oatmeal tomato agar (OTA) plates at 5 dpi. (**B**) Colony diameters of strains P131, MUG1ko1, MUG2ko1, MUG3ko1, and MUG4ko1 in (**A**). The means and standard deviations were calculated from three replicates. (**C**) Capacity for conidiation of strains P131, MUG1ko1, MUG2ko1, MUG3ko1, and MUG4ko1 on an OTA plate. The means and standard deviations were calculated from three replicates. Asterisks indicate a significant difference in conidiation between strains MUG1ko1 and P131 at *p* < 0.01 according to a *t*-test. (**D**) Conidiation structure of strains P131, MUG1ko1, MUG1ko2, MUG1com1, and MUG1com2. Co, conidium. Cp, conidiophore. Bar, 20 μm.

The asexual development of the deletion mutants of the *MUG* genes was assayed by culturing the strains on OTA plates. As expected, there were no differences between these *mug* deletion mutants and strain P131 (Figure 5A,B). Nevertheless, the *mug1* deletion mutants, but not others, exhibited defects in conidiation and produced 0.9 × 10^7^ conidia per plate, which was about two-thirds of that of the wild-type strains (Figure 5C). Moreover, strains MUG1ko1 and MUG1ko2 produced fewer conidiophores with no more than 4 conidia on each conidiophore, while strain P131 produced abundant conidiophores and 3–10 conidia per conidiophore (Figure 5D). These findings suggest that *MUG1* plays an important role in conidiation.

## 4. Discussion

In pathogenic fungi, species-unique genes with high expression levels during the infection stage are considered to be important for fungal virulence. The *Fusarium*-unique gene *OSP24* is highly expressed during the infection of wheat heads and is required for IH development and fungal virulence [2]. The *Magnaporthe*-unique gene *MoHKR1* was also highly expressed during rice leaf infection and is required for IH growth and fungal virulence [4]. Similar to *MoHKR1*, another *Magnaporthe*-unique gene, *MUG1*, reported in this study, was also required for appressorial turgor generation, IH development, and fungal virulence. Consequently, it will be interesting to investigate the underlying mechanisms of how these species-unique genes regulate fungal virulence by uncovering their interacting proteins and signaling pathways.

Genes whose deletion cause minor roles in fungal virulence and asexual development might contribute to adapting to complicated external environments. For example, a combination of the transcriptome and proteome of an obligate psychrophilic fungus *Mrakia psychrophile,* treated at 4, 12, and 20 °C, indicated that 469 of 1276 *Mrakia*-unique genes were differentially expressed, suggesting their important roles in adapting to cold stress [39]. In *M. oryzae*, the deletion of individual species-unique genes *MoSPC1*, *MoSPC2*, *MoSPC3*, and *MoSPC7* caused no detected phenotypes on fungal pathogenicity [1]. The deletion of the species-unique gene *MIR1*, which is specially expressed during plant infection, also causes no detectable changes in fungal pathogenicity [21]. Thus, we speculate that these species-unique genes including the three in this study, *MUG2*, *MUG3*, and *MUG4,* might be important for the fitness and virulence of *M. oryzae* under field conditions.

In contrast to avirulence proteins specific to a subset of isolates of a certain species, species-unique proteins are highly conserved within the species. Hence, fungal virulence- or pathogenicity-essential species-unique proteins are supposed to be ideal targets for developing green fungicides. If active chemical compounds are developed by targeting unique domains of motifs in these proteins, they should be very effective at inhibiting the infection by a specific fungus and have non-toxic side effects on other fungal species, plants, and animals [4,33]. For example, the *Magnaporthe*-unique effector protein MoErs1 is indispensable for fungal virulence by inhibiting the function of rice papain-like cysteine protease OsRD21 involved in rice immunity and an active small chemical FY21001, which specifically binds to and inhibits MoErs1 function and significantly and effectively controls rice blast [40]. The *Rhizoctonia cerealis*-unique effector protein RcOSP1 impairs wheat biological processes and suppressed pathogen-associated molecular pattern-triggered immunity during the fungal infection process; silencing RcOSP1 can inhibit fungal growth through host and spray-induced gene silencing approaches [41]. Therefore, the identification of fungal virulence- or pathogenicity-essential species-unique proteins will open up an avenue for identifying novel fungicide-target proteins.

In summary, in this study, we functionally characterized four *Magnaporthe*-unique genes and found that *MUG1* plays a vital role in infection-related morphogenesis and conidiation in rice blast fungi, which might be a potential target for developing green fungicides. Our findings also highlight the specific and important roles of species-unique genes in fungal pathogenesis and asexual development.

## Data Availability

The original contributions presented in the study are included in the article/Supplementary Materials, further inquiries can be directed to the corresponding author.

## Supplementary Materials

The following supporting information can be downloaded at https://www.mdpi.com/article/10.3390/jof10080511/s1. Figure S1: Gene deletion of (A) *MUG1*, (B) *MUG2*, (C) *MUG3*, and (D) *MUG4*; Table S1: Primers used in this study.
