# Supplementary material for: Magnaporthe-Unique Gene MUG1 Is Important for Fungal Appressorial Penetration, Invasive Hyphal Extension, and Virulence in Rice Blast Fungi
Source: J Fungi (Basel). 2024 Jul 23;10(8):511. doi: 10.3390/jof10080511 (PMC11355306; doi:10.3390/jof10080511)
Supplement: Supplementary file 1 [file jof-10-00511-s001.zip › jof-3100485-supplementary.pdf]

Supplementary material

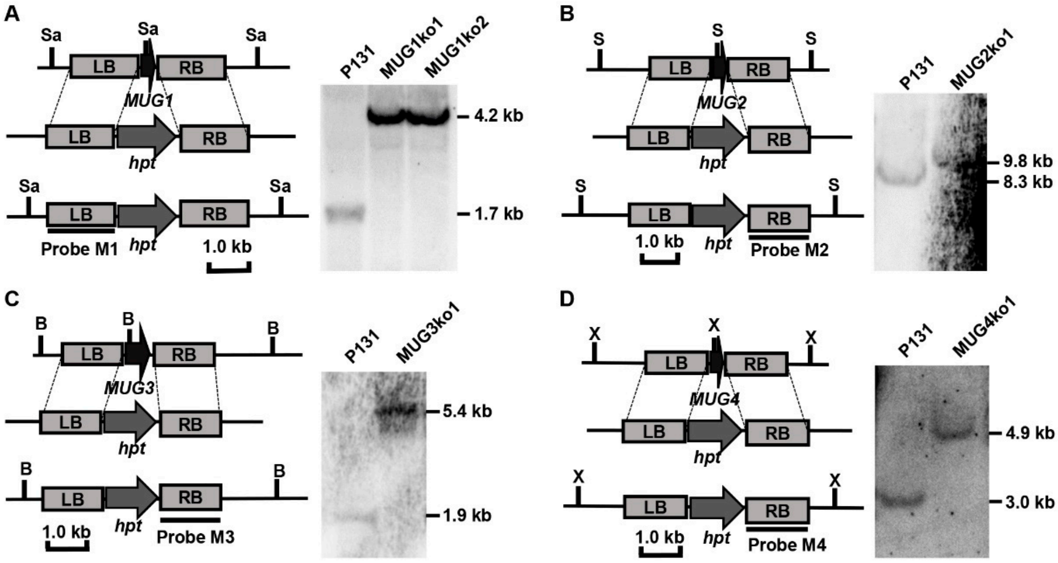

Figure S1 Gene deletion of *MUG1*, *MUG2*, *MUG3*, and *MUG4*. (A) Schematic diagram of the *MUG1* deletion strategy and Southern blot analysis of *SalI* (Sa)-digested genomic DNA of wild-type P131 and *MUG1* gene-deletion mutants MUG1ko1 and MUG1ko2 hybridized with probe M1. The estimated size of each band was labeled on the right (kb). *hpt*, hygromycin phosphotransferase gene. (B) Schematic diagram of the *MUG2* deletion strategy and Southern blot analysis of *SacII* (S)-digested genomic DNA of P131 and the *MUG2* gene-deletion mutant MUG2ko1 hybridized with probe M2. (C) Schematic diagram of the *MUG3* deletion strategy and Southern blot analysis of *BamHI* (B) digested genomic DNA of P131 and the *MUG3* gene deletion mutant MUG3ko1 hybridized with probe M3. (D) Schematic diagram of the *MUG4* deletion strategy and Southern blot analysis of *XhoI* (X)-digested genomic DNA of P131 and *MUG4* gene deletion mutant MUG4ko1 hybridized with probe M4.

Table S1 Primers used in this study.

Primer	Sequence (5’→3’)
MUG1-LB-F	AGAACTAGTGGATCCCCCGGGCAACACTTACCTACGGCTG
MUG1-LB-R	TCAATATCAGTTATCGAATTCGAGATGGGAGAGGAAGAGAAA
MUG1-RB-F	CCAGTTATCAAGCTTATCGATGCGGACGCTACTACTGAAA
MUG1-RB-R	AGGGAACAAAAGCTGGGTACCGAAATACGGACCCTTGGAA
MUG1-check-F	GAAGAGGGAGGTTACAGACG
MUG1-check-R	TTCAAAGCACCGAGACATC
MUG1-gene-F	CGTTTCGAACTCCCCATTATC
MUG1-gene-R	TAACCTTGCTTTGCCGTC
MUG1-Q-F	CAGACTGCCGACTACCACTC
MUG1-Q-R	CCATCTGCCTCTTTTGGTGG
Mug1-GFP-F	AAGCTTGATATCGAATTC TGTAAGCCAGAGTGCAACC
Mug1-GFP-R	TCTAGAACTAGT GGATCCTG AACGGTATGACGCGTG
MUG2-LB-F	AGAACTAGTGGATCCCCCGGGCACAGTCAGGACATTTCGG
MUG2-LB-R	TCAATATCAGTTATCGAATTCGGAGTTTCGCAGCAATCAC
MUG2-RB-F	AAGCTTATCGATACCGTCGACCGTTGGAGTGTTTCTGGAA
MUG2-RB-R	GGTACCGGGCCCCCCCTCGAGGGGCGTAAATGGATGTGAC
MUG2-check-F	CCTTTGATGAGTCGTTTGC
MUG2-check-R	ATAGTGAAAGAGGGCAGGG
MUG2-gene-F	ATGAAGATGG CATCATCGGC
MUG2-gene-R	AGCAGGGAAG GTAGACGTCG
MUG2-Q-F	GGCCGACATCAACCATTCAG
MUG2-Q-R	CCCATTCCATTCCAAGCCTG
Mug2-GFP-F	AAGCTTGATATC GAATTC TCGCTTGTAC CTAGGTCTAC
Mug2-GFP-R	TCTAGAACTAGT GGATCC GCCGGCAGGGCGATCGAGGC
MUG3-LB-F	GGCGGCCGCTCTAGAACTAGTCACACAGTCCGGGTCTTTTG
MUG3-LB-R	TCAATATCAG TTATCGAATTCGGCTATGCTGAATCTTGGCT
MUG3-RB-F	CCAGTTATCATCGATCGTGATTGGCGGGACTAAT
MUG3-RB-R	GGTACCGGGCCCCCCCTCGAGTCACCCTGTCAAAGAACTCA

MUG3-check-F	TTGAAGGGCGACAAGGAT
MUG3-check-R	GCATGTAATAAGTAGCCGT
MUG3-gene-F	ATGCAATTTA CACACAGTAA
MUG3-gene-R	CAGCACAATT GAATGTGGTA
MUG3-Q-F	AAACCAACACCAAAGGCTGC
MUG3-Q-R	GAGAGGGCATGGGATCTGAA
Mug3-GFP-F	AAGCTTGATATC GAATTC TCACCAGCAG GCGAGTCCAA
Mug3-GFP-R	TCTAGAACTAGT GGATCC TGCAAAACCT ATCCGCCCCG
MUG4-LB-F	GGCGGCCGCTCTAGAACTAGTCACACGATTTCTCACCCCTT
MUG4-LB-R	TCAATATCAGTTATCGAATTCACAGAAACCTCCGAGCAA
MUG4-RB-F	CGGGAACCAGTTATCAAGCTTCCCAGCATCACTTGTAATCA
MUG4-RB-R	CTCGAGGTCGACGGTATCGATGCAACGCATCGGTAAAGA
MUG4-check-F	CGTAGTAATCGCCATTGGG
MUG4-check-R	ACCTGTTCCACCACGACTGT
MUG4-gene-F	GGTCGATCAACGTTCCCTAC
MUG4-gene-R	AGAACCCTGTGCTTCCTGT
MUG4-Q-F	TCGCCTTTTGATCTCCTTGC
MUG4-Q-R	AGAACCCTGTGCTTCCTGTA
Mug4-GFP-F	AAGCTTGATATC GAATTC GTCATCGCAA CACACGATTT
Mug4-GFP-R	TCTAGAACTAGT GGATCC CCGACGCAGG TTCAGAACCC
HPT-up	TCTGACCAGTTGCCTAAATG
HPT-down	ACCGATGGCTGTGTAGAAG
